# Genome Sequencing and Analysis of the Fungal Symbiont of Sirex noctilio, Amylostereum areolatum: Revealing the Biology of Fungus-Insect Mutualism

**DOI:** 10.1128/mSphere.00301-20

**Published:** 2020-05-13

**Authors:** Ningning Fu, Ming Wang, Lixiang Wang, Youqing Luo, Lili Ren

**Affiliations:** a Beijing Key Laboratory for Forest Pest Control, Beijing Forestry University, Beijing, China; b Sino-French Joint Laboratory for Invasive Forest Pests in Eurasia, Beijing Forestry University—INRAE, Beijing, China; University of Wisconsin—Madison

**Keywords:** secondary metabolism, *Amylostereum areolatum*, *Sirex noctilio* (F.), cell wall-degrading enzymes, genome, virulence

## Abstract

Amylostereum areolatum is the symbiotic fungus of the Eurasian woodwasp, Sirex noctilio, a globally invasive species. The mutualistic symbiont is associated with the woodwasp, assisting the damage process and providing nutrition for its insect partners. Colonization and growth of A. areolatum have essential impacts on the development and spread of S. noctilio, though the mechanism of interaction between the two has been poorly described. In this study, the first genome of this symbiotic fungus was sequenced, assembled, and annotated. The assembled A. areolatum genome was 57.5 Mb (54.51% GC content) with 15,611 protein-coding genes. We identified 580 carbohydrate-active enzymes (CAZymes), 661 genes associated with pathogen-host interactions, and 318 genes encoding transport proteins in total. The genome annotation revealed 10 terpene/phytoene synthases responsible for terpenoid biosynthesis, which could be classified into three clades. Terpene synthase gene clusters in clade II were conserved well across Russulales. In this cluster, genes encoding mevalonate kinase (MK), EGR12 (COG1557), and nonplant terpene cyclases (cd00687) were the known biosynthesis and regulatory genes. Genome sequence analysis of this fungus would prove the possibility of A. areolatum volatiles affecting the host selection of S. noctilio on a molecular basis. We further clarified that A. areolatum was a strict obligate symbiotic fungus. The wasps might protect the fungus before it was introduced into a suitable host substrate by oviposition, while the fungus would provide S. noctilio with a suitable environment and nutrients for the larval growth. These results would lay a foundation for our understanding of the mechanism of this entomogenous symbiosis.

**IMPORTANCE** Sirex noctilio (F.), together with Amylostereum areolatum, a wood-decaying symbiotic fungus, causes severe damage to *Pinus* species worldwide. In China, it causes extensive death of Mongolian pine (Pinus sylvestris var. *mongolica*). There is an obligate dependency mutualism between the woodwasp and its fungus. Studies have suggested that the fungal growth rate affected the size of the wasps: larger adults emerged from sites with a higher fungus growth rate. This genome is the first reported genome sequence of a woodwasp symbiotic fungus. Genome sequence analysis of this fungus would prove the possibility of A. areolatum volatiles affecting the host selection of S. noctilio on a molecular basis. We further clarified that A. areolatum was a strict obligate symbiotic fungus and that it would provide S. noctilio with a suitable environment and with nutrients for the larval growth. These results would lay a foundation for our understanding of the mechanism of this entomogenous symbiosis.

## Abstract (precis)

Sirex noctilio (F.), together with Amylostereum areolatum, a wood-decaying symbiotic fungus, causes severe damage to *Pinus* species worldwide. In China, it causes extensive death of Mongolian pine (Pinus sylvestris var. *mongolica*). There is an obligate dependency mutualism between the woodwasp and its fungus. Studies have suggested that the fungal growth rate affected the size of the wasps: larger adults emerged from sites with a higher fungus growth rate. This genome is the first reported genome sequence of a woodwasp symbiotic fungus. Genome sequence analysis of this fungus would prove the possibility of A. areolatum volatiles affecting the host selection of S. noctilio on a molecular basis. We further clarified that A. areolatum was a strict obligate symbiotic fungus and that it would provide S. noctilio with a suitable environment and with nutrients for the larval growth. These results would lay a foundation for our understanding of the mechanism of this entomogenous symbiosis.

## INTRODUCTION

The Eurasian woodwasp, Sirex noctilio (F.) (Hymenoptera; Symphyta; Siricidae), is an exotic invasive pest that damages *Pinus* species. It is recognized as a high-risk invasive species by the North American Plant Protection Organization (NAPPO) and the United States Department of Agriculture (USDA) (1, 2). S. noctilio is native to Europe and North Africa, where woodwasp is generally considered a secondary pest and of negligible economic concern. However, it had spread and established in several countries in the Southern Hemisphere during the twentieth century and, later, in North America and Southern Africa (3–6). In China, it was first found in Daqing, Heilongjiang Province, in 2013, and has been spotted in many cities since then, causing substantial deaths in Mongolian pine (Pinus sylvestris var. mongolica) plantations (7).

Amylostereum areolatum (Fr.) Boidin (Basidiomycotina: Corticiaceae) is a fungal symbiont of S. noctilio. There is a very strict obligate dependency mutualism between woodwasp and its fungal symbiont A. areolatum (7). S. noctilio has a specialized organ for storing symbiotic fungus, the mycangium, that is a reliable indicator of reciprocal, mutually beneficial adaptations and, in many cases, of obligate dependencies (2). First, the mycangium protects the fungus before it is introduced into a suitable host substrate. Next, during oviposition, the female woodwasp inoculates its symbiotic fungus and phytotoxic venom mucus into the host tree with the egg through the ovipositor. Sometimes, when the host is not suitable, no egg is deposited and only the fungus and venom are (8–10). Starting from the second instar, the fungus migrates from the previous larval instar to the next. When the adult emerges, the fungus is taken up from the wall of the pupal chamber into its mycangium (7). In this way, the female collects the oidia of A. areolatum produced in the insect galleries for dissemination and inoculation into new trees. In turn, the fungus is also essential to the development and even the reproductive potential of S. noctilio (11–14). A. areolatum infects and dries the wood substrate to provide a more suitable microenvironment for egg and larvae. In addition, degradation of cellulose, hemicellulose, and pectin by rich repertoire enzymes of the symbiotic fungus is presumably crucial for the woodwasp larvae. The development of the woodwasp, and thus its reproductive potential, are tied to the vigor of its symbiotic fungus (2). Studies have shown that woodwasp larvae feed on A. areolatum until the third or fourth instar and then feed on white-rotted wood (14–16). Madden and Coutts (1979) suggested that the fungus growth rate affected the size of the woodwasps; larger adults emerged from sites with higher rates of fungus growth (12). Obviously, the woodwasp derives considerable benefits from its partnership with A. areolatum, and the benefits are extensively linked to the colonization and growth of the fungi in pine hosts. Carbohydrate-active enzymes (CAZymes) and virulence-related genes are essential in the colonization and growth of fungus. White rot fungi can efficiently degrade lignocellulosic biomass, especially that derived from plants, for their diverse CAZymes (17). Many virulence-related genes that have been studied were found to be associated with the manipulation of plant defenses to promote fungus infections (18). These genes play an important role in defense mechanisms, signal transduction, carbohydrate transport and metabolism, intracellular trafficking, secretion, and vesicular transport (18–20).

Volatile components play an important role in the attraction of female woodwasps to the plant host. A Y-tube olfactometer was used to assess the behavior preferences of adult female wasps with respect to volatiles (21). Previous research showed that S. noctilio showed a stronger positive response to volatile components of A. areolatum than to those of attacked pines. These fungal volatiles could also attract woodwasps to hosts infected by A. areolatum (21). Our laboratory analyzed the volatile organic compounds (VOCs) of A. areolatum and other endophytic fungi in Mongolian pine and found that sesquiterpenes attract mated S. noctilio females but not unmated ones (15). It may be more important for a mated female to locate a weakened tree that has been inoculated with the fungi needed for the development of the woodwasp (22, 23). Although terpenoid compounds may play an important role in spatial aggregation of woodwasps, no terpene biosynthetic enzyme has so far been described in A. areolatum. Genome sequencing methods are commonly used to discover biosynthetic pathways of terpenoid compounds by identifying the genes involved in the activity of secondary metabolites. For example, the potential terpene synthase (TPS) genes in the wood-rotting fungi Stereum hirsutum and Coprinus cinereus were identified by genome sequencing, and their biochemical activities were subsequently characterized (24, 25). Similarly, the availability of genome sequences may lead to the discovery of the TPS gene family in A. areolatum.

Wood-feeding insects are facing many difficulties in obtaining nutrition from their host’s wood resources. The European woodwasp is associated with its mutualistic fungus, A. areolatum, which assists host selection and provides nutrition for its insect partner, though the interaction mechanism for this symbiosis has been poorly described. In this study, we completed the genome sequencing of the symbiotic fungus of S. noctilio and analyzed carbohydrate enzymes, virulence genes, and secondary metabolism genes. These findings would help us to explain the hypothesis that there are enzymes secreted by A. areolatum playing crucial roles in supporting the life cycle of S. noctilio, such as by degrading plant cell walls to provide nutrients for the colonization and growth of S. noctilio larvae and by producing sesquiterpene compounds to attract the female to oviposit. Our results lay a foundation for a better understanding of the mechanism of mutualism between S. noctilio and A. areolatum.

## RESULTS

### General genomic characteristics.

**FIG 1:**
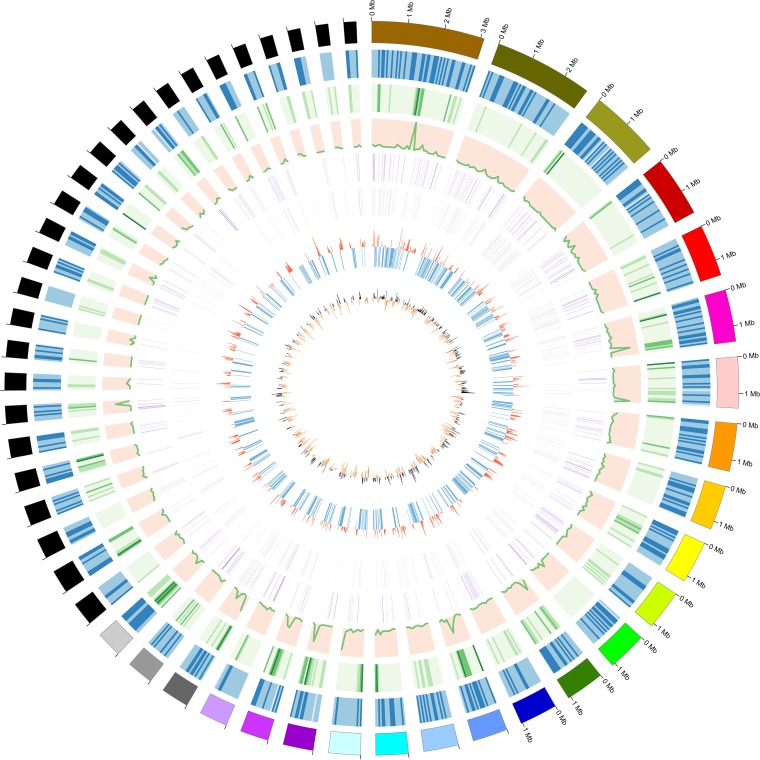
Circular representation of genomic features of A. areolatum. The graph shows the genomic features of Amylostereum areolatum (Fr.) Boidin. The circles illustrate, from outside to inside, the scaffolds (sorted by length) of A. areolatum. The second and third circles show gene densities (gene number in 50,000-bp nonoverlapping windows) and repetitive sequences (repeat coverage length in 50,000-bp nonoverlapping windows). The fourth circle shows ncRNA densities (ncRNA number in 100,000-bp nonoverlapping windows). The fifth circle shows genes coding for CAZy proteins. The sixth circle contains members of the PHI gene group. The seventh and last circle indicates percent G+C content and GC skew (in 20,000-bp nonoverlapping windows).

The A. areolatum genome was sequenced using a combination of Illumina and PacBio Sequel technologies. After quality control, we obtained 6,839 Mb of HiSeq data (118× coverage) and 4,314 Mb of PacBio data (74× coverage). Combined sequences from the two platforms were assembled into 248 scaffolds with an *N*_50_ value of 789 kb to obtain a total genome size of 53,481,184 bp (54.51% GC content) (Fig. 1) (Table 1). In addition, we predicted 15,611 genes, with an average length of 2,275 bp (Table 1). Among the protein-coding genes, 13,378 (85.69%) had significant sequence similarity to previously documented fungal sequences, whereas 14.31% of the genes did not have significant matches to any known genes. BUSCO was used to calculate the completeness of assembly and annotation. Among 290 single-copy orthologs, 92.8% of contigs were complete (232 complete single-copy BUSCOs and 37 complete duplicated BUSCOs), while only 1.4% were fragmented and 5.8% were missing.

**TABLE 1:** Whole-genome assembly features of *Amylostereum areolatum* (Fr.) Boidin

Parameter or annotation	Value
Assembly parameters	
Total genome size (bp)	57,567,124
No. of scaffolds	248
Maximum scaffold length (bp)	3,099,494
Minimum scaffold length (bp)	365
Depth of genome coverage (×)	118
Scaffold *N*_50_ length (bp)	731,813
GC content (%)	54.51

Gene annotations	
Total no. of genes	15,611
Total no. of annotated genes	13,378
Total no. of ncRNAs	722

In further analyses, we detected 97,258 exons with an average length of 245.35 bp (total length, 35,517,529 bp). The average length of the introns was 142.76 nucleotides. For noncoding RNAs (ncRNAs), 457 tRNA genes in the A. areolatum genome were identified using tRNAscan-SE (11). Among the tRNAs, 429 anticodon tRNAs corresponded to the 20 common amino acids and the other tRNAs represented possible pseudogenes.

### Repetitive elements and transposases.

Besides protein-coding gene sequences, an important portion of the fungal genome is the repetitive element. In the A. areolatum genome, we identified 14,513,406 bp (25.21% of the genomic sequence) of repetitive elements (Fig. 1; see also Table S1 in the supplemental material). Tandem repeat sequences accounted for 1.1% and transposable elements (TEs) for 24.11% of the assembled genome. Among the TEs, class I element (retrotransposon) TEs and class II element (DNA) TEs accounted for 15.50% and 1.36% of the genome, respectively. Unknown and other repetitive elements covered 8.17%. Long terminal repeats (LTRs) were the most abundant class I TEs (14.79% of the genomic sequence) and included the Copia, Gypsy, and BEL/Pao retrotransposon superfamilies.

10.1128/mSphere.00301-20.4 TABLE S1 Repeat sequence annotation of *A. areolatum*. Download Table S1, XLSX file, 0.01 MB. Copyright © 2020 Fu et al. 2020 Fu et al. This content is distributed under the terms of the Creative Commons Attribution 4.0 International license.

### Phylogeny.

**FIG 2:**
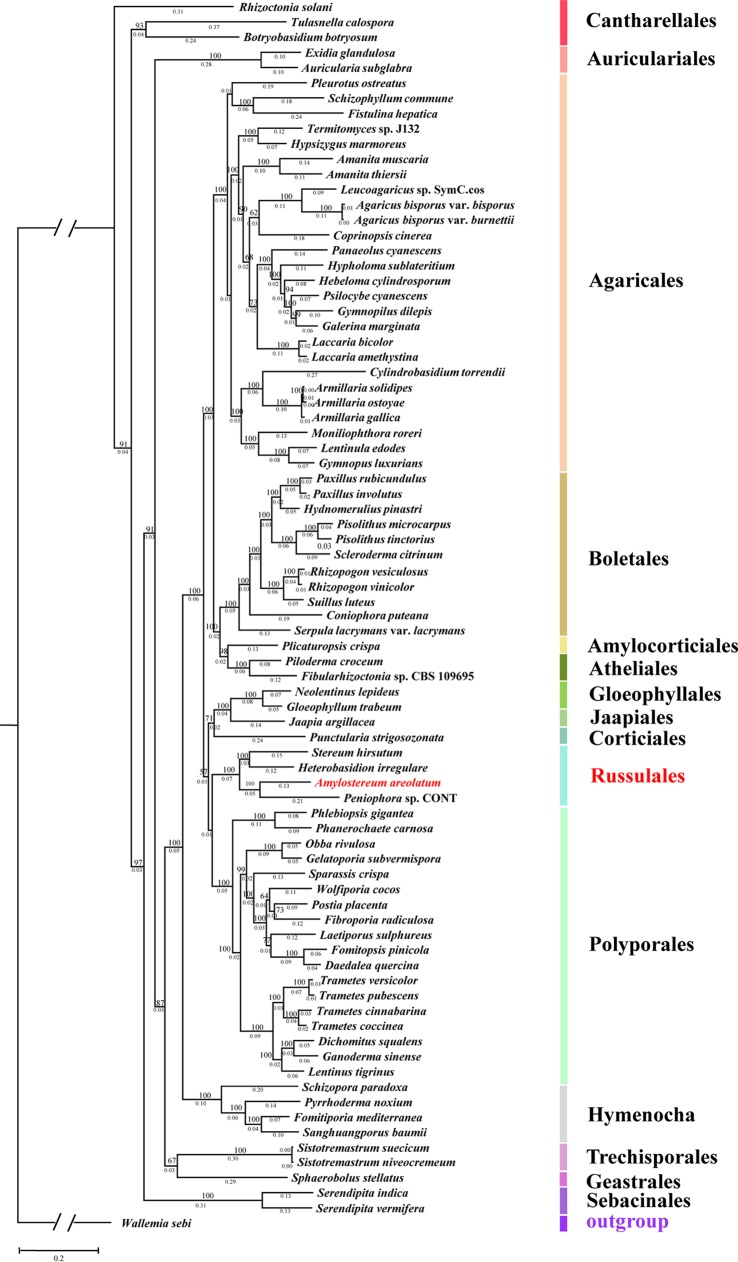
Phylogenetic location of A. areolatum among Agaricomycetes. The topology of the phylogenetic tree was constructed by the maximum likelihood method (bootstrap = 1,000, LG+I+G+F model); A. areolatum is shown in red. Bootstrapping-based branch support and a scale bar representing the mean number of amino acid substitutions per site are shown.

OrthoFinder identified 56,374 gene clusters, among which were included 913 orthologous genes shared among all the fungal species. From these shared gene clusters, 41 single-copy orthologous genes were chosen to analyze the evolutionary relationship of A. areolatum and other 82 Agaricomycetes reference genomes (Table S2). The maximum likelihood (ML) analysis identified, with high (>70%) bootstrap support, 15 major fungal clades of the orders Cantharellales, Auriculariales, Agaricales, Boletales, Amylocorticiales, Atheliales, Gloeophyllales, Jaapiales, Corticiales, Russulales, Polyporales, Hymenochaetales, Trechisporales, Geastrales, and Sebacinales (Fig. 2). Phylogenetic analysis revealed that A. areolatum clustered with other Russulales species and close to the plant-pathogenic fungus *Peniophora* sp. strain CONT.

10.1128/mSphere.00301-20.5 TABLE S2 Reference genomes for comparative analysis. Download Table S2, XLSX file, 0.01 MB. Copyright © 2020 Fu et al. 2020 Fu et al. This content is distributed under the terms of the Creative Commons Attribution 4.0 International license.

### Carbohydrate-active enzymes.

**FIG 3:**
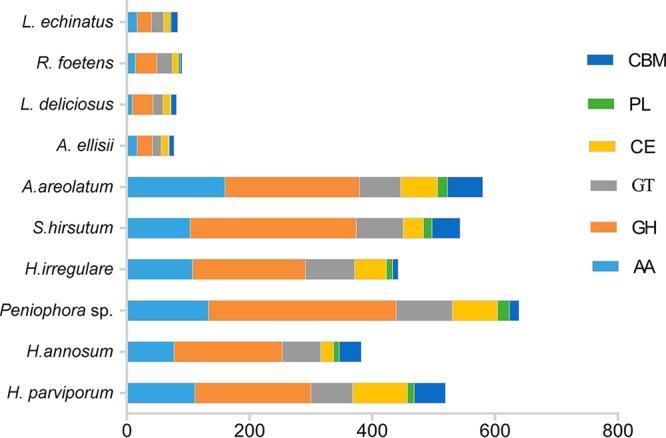
Total CAZymes from A. areolatum and other fungal species. AA, auxiliary activities; GH, glycoside hydrolase; GT, glycosyltransferase; CBM, carbohydrate-binding module; PL, polysaccharide lyase. L. echinatus, Lactarius echinatus; R. foetens, Russula foetens; L. deliciosus, Lactarius deliciosus; A. ellisii, Albatrellus ellisii; A. areolatum, Amylostereum areolatum; S. hirsutum, Stereum hirsutum; H. irregulare, Heterobasidion irregulare; Peniophora sp., Peniophora sp. strain CONT; H. annosum, Heterobasidion annosum; H. parviporum, Heterobasidion parviporum.

In the A. areolatum genome, we identified 580 CAZymes, consisting of 219 glycoside hydrolases (GHs), 67 glycosyl transferases (GTs), 16 polysaccharide lyases (PL), 60 carbohydrate esterases (CEs), 58 carbohydrate-binding modules (CBMs), and 160 enzymes with auxiliary activities (AAs) (Table S3). While the number of CAZymes in A. areolatum (580) was comparable to the number in other Russulales fungi, the number of CAZyme genes was comparable to the number in the forest pathogens Heterobasidion parviporum (519), S. hirsutum (543), Heterobasidion irregulare (442), and *Peniophora* sp. strain CONT (639). A. areolatum had more CAZyme genes than the traditional saprophytic mushrooms Lactarius echinatus (83), Russula foetens (90), Lactarius deliciosus (81), and Albatrellus ellisii (77) (Fig. 3).

10.1128/mSphere.00301-20.6 TABLE S3 Number of CAZy proteins. Download Table S3, XLSX file, 0.02 MB. Copyright © 2020 Fu et al. 2020 Fu et al. This content is distributed under the terms of the Creative Commons Attribution 4.0 International license.

There were more genes encoding GHs and AAs than other protein types in the A. areolatum genome. We found that the members of 45 GH gene families, including GH3 (15 copies), GH5 (18 copies), and GH18 (16 copies), were particularly abundant. In addition, we found two GH6 and eight GH7 members related to the degradation of crystalline cellulose. A classification of the AA family revealed that a majority of the AAs were members of the AA3 family (62 copies), including the subfamilies of cellobiose dehydrogenase (EC 1.1.99.18), glucose 1 oxidase (EC 1.1.3.4), aryl alcohol oxidase (EC 1.1.3.7), alcohol oxidase (EC 1.1.3.13), and pyranose oxidase (EC 1.1.3.10) (Fig. 1) (Table S3).

### Putative virulence-associated genes.

**FIG 4:**
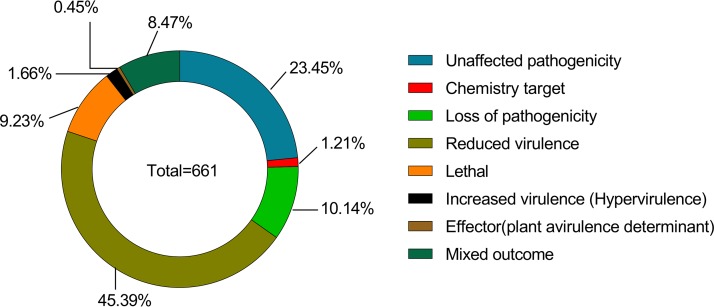
Predicted phenotypic-category genes in A. areolatum. The distribution of phenotypic categories of A. areolatum gene orthologs was determined using the PHI database; percentages are based on 661 hits.

We found a total of 661 predicted A. areolatum genes associated with pathogen-host interactions (PHI). The highest proportion (45.39%) was related to “reduced virulence,” followed by “unaffected pathogenicity” (23.45%), “loss of pathogenicity” (10.14%), “lethal” (9.23%), “mixed outcome” (8.47%), “chemistry target” (1.21%), “increased virulence (hypervirulence)” (1.66%), and “effector (plant avirulence determinant)” (0.45%) (Fig. 1; see also Fig. 4) (Table S4). Besides, we annotated 47 genes that have been reported as virulence factors (VFs) in pathogenic bacteria (virulence factors of pathogenic bacteria [Virulence Factor Database {VFDB}]) (Table S5). These genes may be putative pathogenicity factors in A. areolatum based on their established role in pathogenesis in other host species. Furthermore, we identified 318 transport proteins belonging to 83 families in A. areolatum. Among these transporters, 37 genes encoded major facilitator superfamily (MFS) proteins and 18 encoded ATP-binding cassette (ABC) proteins; fewer genes encoded proteins in other families (see Fig. S2 in the supplemental material; see also Table S6).

10.1128/mSphere.00301-20.7 TABLE S4 Number of candidate pathogenicity genes. Download Table S4, XLSX file, 0.03 MB. Copyright © 2020 Fu et al. 2020 Fu et al. This content is distributed under the terms of the Creative Commons Attribution 4.0 International license.

10.1128/mSphere.00301-20.8 TABLE S5 Number of virulence factors. Download Table S5, XLSX file, 0.01 MB. Copyright © 2020 Fu et al. 2020 Fu et al. This content is distributed under the terms of the Creative Commons Attribution 4.0 International license.

10.1128/mSphere.00301-20.9 TABLE S6 Number of transporters. Download Table S6, XLSX file, 0.01 MB. Copyright © 2020 Fu et al. 2020 Fu et al. This content is distributed under the terms of the Creative Commons Attribution 4.0 International license.

### Sesquiterpene synthase clusters.

AntiSMASH analysis revealed that the number and type of secondary metabolite genes predicted for A. areolatum were comparable with those reported for other Russulales species, which carried 19 secondary metabolism gene clusters, including clusters of genes encoding eight terpene/phytoene synthases, two nonribosomal peptide synthetases (NRPS), one type-I polyketide synthase (T1PKS), and one siderophore synthase and seven unknown gene clusters (Table S7). It is worth mentioning that we identified the largest number of terpene/phytoene synthase gene clusters in the genome of A. areolatum. These enzymes might be involved in the biosynthesis of a terpenoid, although further investigation is needed to ascertain this.

10.1128/mSphere.00301-20.10 TABLE S7 Number of secondary metabolism genes in the genomes of Russulales. Download Table S7, XLSX file, 0.01 MB. Copyright © 2020 Fu et al. 2020 Fu et al. This content is distributed under the terms of the Creative Commons Attribution 4.0 International license.

**FIG 5:**
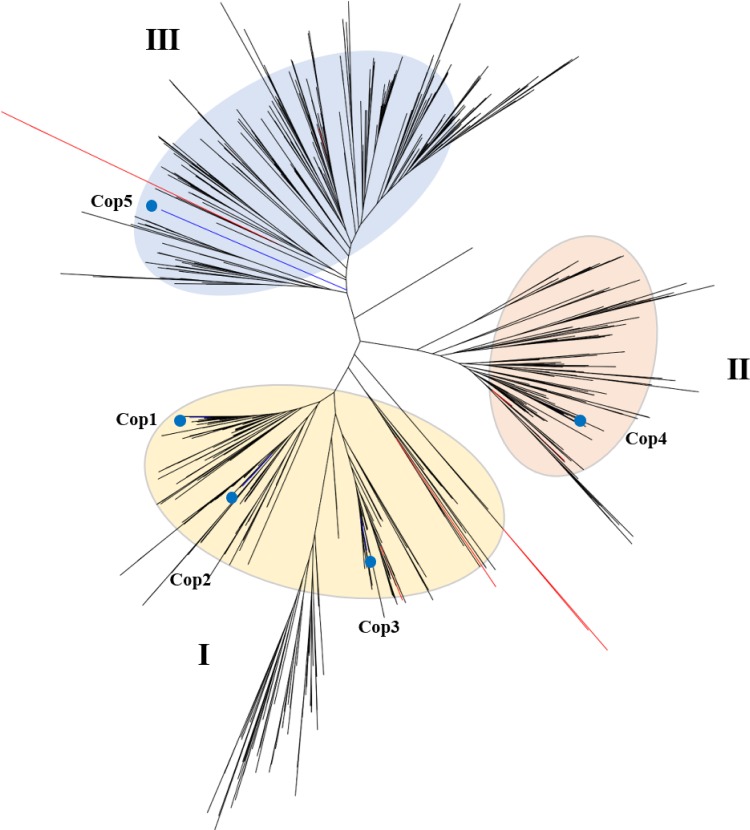
Phylogram of terpene synthase homologs identified in 83 Agaricomycetes genomes. We used 902 putative terpene synthase genes from two orthologous groups, in which five sesquiterpene synthase homologs (named Cop1 to Cop5) identified in C. cinereus were included. The red and blue branches represent sesquiterpene synthase of A. areolatum and C. cinereus, respectively.

To elucidate the conserved and diverged structures of the terpene biosynthetic clusters, we obtained two ortholog groups containing five sesquiterpene synthase (STS) genes identified in C. cinereus from the 83 Agaricomycetes genomes. The A. areolatum genome contained 10 terpene synthase genes, consistent with the number of such enzymes in Agaricomycetes. A phylogenetic tree was constructed using the orthologs of three well-characterized C. cinereus terpene synthases, Cop3, Cop4, and Cop5, that were identified in the A. areolatum genome (Fig. 5). Terpene synthase genes of A. areolatum clustered with the Cop STS genes into three major groups, suggesting that the enzymes in each cluster might produce the related terpenoid compounds through conserved cyclization pathways (Fig. S3).

**FIG 6:**
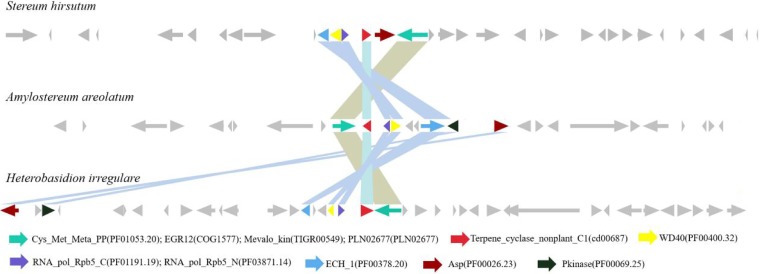
Synteny of terpenoid gene cluster among A. areolatum, S. hirsutum, and H. irregulare.

In fungi, secondary metabolite biosynthetic genes usually locate in contiguous clusters (24). Interestingly, terpene synthase gene clusters in clade II were conserved well across Russulales. Synteny among A. areolatum, S. hirsutum, and H. irregulare showed that genes involved in the synthesis of terpenoid compounds had good collinearity, indicating that Russulales might share conserved and core genes related to terpene metabolism (Fig. 6). The well-conserved gene cluster contained mevalonate kinase (MK), EGR12 (COG1557), nonplant terpene cyclases (cd00687), and enoyl-coenzyme A (enoyl-CoA) hydratase/isomerase and pkinase, in which some genes have been proven to be related to the mevalonate pathway (one of the pathways functioning to synthesize terpenoids). In addition to the core terpene cyclases involved in sesquiterpene metabolism, ABC transporter ATP-binding protein (CL0023) and P450 (PF00067) were also present in the sesquiterpene clusters. These enzymes might play a role in modifications such as oxidation and hydroxylation, as well as in transportation of sesquiterpene.

## DISCUSSION

### General genomic features and phylogeny.

The genome assembly was about 53 Mb, comparable to the size of assemblies for the related members of Russulales, which range from 26 Mb to 122 Mb (26–30). The A. areolatum genome contained 15,611 coding genes, similar to the number of coding genes in Russulales fungus S. hirsutum (26) (14,066), *Peniophora* sp. strain CONT (30) (18,945), and H. irregulare (27) (13,275). This indicated that the genome assembly of A. areolatum was comparable in fragmentation and continuity to the other high-quality white rot fungal genomes. The phylogenetic tree showed that A. areolatum was evolutionally close to the plant-pathogenic fungus *Peniophora* sp. strain CONT rather than to S. hirsutum (fungi belonging to the same family as A. areolatum). Another study analyzing the phylogenetic relationship of Russulales species on the basis of sequences of the nuc ribosomal DNA (rDNA) ITS1-5.8S-ITS2 and D1-D2 domains of nuc 28S rDNA showed that *Amylostereum* belonged to the family Echinodontiaceae (31). This result may have been due to the limited availability of Russulales resources and the lack of genomes of species with relatively close evolutionary relationships. We would likely obtain more detailed results by performing studies with additional genomes of sibling species in the future.

### Protein families involved in degrading plant cell wall and cuticle.

S. noctilio females drill a series of test bores during oviposition to decide whether the trees are suitable for depositing eggs or not, basing on the perceived moisture content and resin pressure of the pine stems (2). All tunnels drilled by females are filled with venom mucus and fungus, but less than half of the tested-suitable tunnels contain eggs. The presence of venom and fungal symbiont in tunnels may cause weakening of hosts and dropping of resin pressure, conditions which provide a more suitable microenvironment for egg hatching and larval development of the woodwasp (2). Besides, the behavioral and morphological adaptations of the woodwasp indicate that S. noctilio larvae do not ingest xylem and that they use the fungi for external digestion of recalcitrant lignocellulosic compounds instead (14). A. areolatum can secrete a large amount of cellulase, hemicellulose, and ligninase, which can effectively degrade the plant cell wall components, providing nutrients (e.g., glucose, mannose, galactose, acetic acid, xylose, etc. [14, 32]) to the larvae of S. noctilio. Therefore, successful colonization and growth of A. areolatum play vital roles in the development of S. noctilio. Plant cell walls represent the main barrier against fungus invasions. CAZymes play a crucial role in the metabolism of glycoconjugates, oligosaccharides, and polysaccharides. They are responsible for the breakdown of cell wall components of hosts to establish a successful infection process. The depolymerizing abilities of the fungi are directly proportional to their ability to secrete a range of CAZymes (33). In our analysis, the number of CAZymes in A. areolatum was comparable to the number in forest pathogens and significantly higher than that in the traditional saprophytic mushrooms, indicating that A. areolatum can break through plant cell walls and successfully establish infection in a manner similar to that seen with other forest pathogens. It can weaken pine trees by destroying the structure of plant cells and provide nutrients for the growth of S. noctilio larvae.

Plant cell walls contain abundant cellulose. GHs are common enzymes that degrade cellulose, hemicellulose, and starch (34). They are involved in the hydrolysis of the glycosidic bond between or within carbohydrate molecules. A total of 219 GHs classified into 45 families were predicted in the A. areolatum genome. Genes encoding GH3 and GH5 class enzymes outnumbered those encoding other GH enzymes. Those were similar to the composition characteristics of GHs in wood-rotting fungi (white, brown, and soft rot fungi) (33). Many enzymes related to degradation of cellulose and xylan belong to the GH3 and GH5 families (35), indicating that this symbiotic fungus can degrade these two substances. In addition, the fungal genome was found to be rich in genes encoding GH18 members, which catalyze the decomposition of chitin. Chitinase can degrade fungal cell walls and inhibit spore germination, mycelial growth, germ tube elongation, and other developmental activities (36, 37). Therefore, it is speculated that GH18 has played a role in the antagonism of other endophytic fungi such as Ophiostoma minus and Chaetomium globosum on Mongolian pine (15). Ninety percent of lignocellulose-degrading fungi contain genes encoding lytic polysaccharide monooxygenases (LPMOs) (38), which are classified into AA families in the CAZy database. These enzymes are mainly involved in the depolymerization of noncarbohydrate structural components (lignin) or are found as primary cell wall components of plants (34, 39). In this study, we detected 62 and 25 copies of AA3 and AA1 in A. areolatum, respectively. AA3 families (GMC oxidoreductases/dehydrogenases) are predominantly found in forest pathogens such as S. hirsutum (48 AA3 genes) and Heterobasidion annosum (32 AA3 genes) (33). The AA3 family contains enzymes from the glucose-methanol-choline (GMC) family of oxidoreductases, which assists in the activity of other AA family enzymes or supports the action of glycoside hydrolases in the degradation of lignocellulose via their reaction products (40). They promote the decomposition of lignocellulose by generating H_2_O_2_ and recycling the electron donors and acceptors required for the oxidative attack of polymers (40).

Laccase is another important lignin-degrading enzyme belonging to the AA1 family. Catalytic oxidation of laccase is involved in the electron capturing of substrates, transfer of electrons between the copper ions in the active center, and, finally, transfer of electrons to O_2_ and reduction of O_2_ to water (41). In fungi, laccases have diverse roles, such as mediation of interactions between fungal pathogens and host plants, stress defense, morphogenesis, and lignin degradation (42, 43). In our study, the high numbers of AA3 and AA1 genes contributed to the oxidative degradation of *Pinus*, which gives A. areolatum a strong ability to penetrate plant cell walls and degrade lignin. This is not only good for fungus colonization and growth, it also provides nutrients to the wasp larvae.

### Virulence-associated genes.

The pathogen-host interactions database (PHI-base) catalogues more than 2,800 genes from fungi, bacteria, and protist pathogens, with experimentally verified pathogenicity, virulence, and effector genes (19, 44, 45). The inactivation or reduction of the expression of these genes can reduce or eliminate pathogenic ability (44). Among all the predicted genes obtained by searches against the PHI-base database, those associated with reduced virulence, unaffected pathogenicity, and loss of pathogenicity were the most frequent in A. areolatum. However, only a few virulence-associated genes (associated with increased virulence [hypervirulence] and effectors [plant avirulence determinants]) were identified. Physiological experiments have shown that inoculation with A. areolatum alone did not significantly reduce tree potential or affect tree growth (46). The characteristics of virulence-related genes of A. areolatum described in our analysis would help explain this phenomenon. Absence of expression or low expression of lethal genes can render the fungus unable to survive (44). There were only 9.23% lethal genes identified in the genome. This might have been because A. areolatum is a typical symbiotic fungus; the wasps would protect the fungus before introducing it into a suitable host substrate. Among the fungal transporters, ATP-binding cassette (ABC) transporters and the major facilitator superfamily (MFS) are the two largest superfamilies. The ABC transporters are multicomponent, primarily active transporters, which transport both small molecules and macromolecules under conditions of ATP hydrolysis (47–49). They transport a broad range of compounds such as polysaccharides, drugs, sugars, heavy metals, oligopeptides, amino acids, and inorganic ions. Studies have shown that, in all sequenced fungi, Schizosaccharomyces pombe contained only 19 ABC proteins, which may be close to the minimal set for a free-living organism (50). A. areolatum has been found to have fewer ABC proteins than S. pombe, and, even more remarkably, it lacks the ABC-D transporters which have been found in every sequenced species except for Encephalitozoon cuniculi and S. pombe (50). Only a few ABC proteins from fungi have been functionally characterized; the lack of ABC-D transporters in A. areolatum needs further study. We speculate that this may be related to the mutualisms of the wasp and fungus and the fact that S. noctilio is a secondary pest that usually colonizes relatively weaker pines (46). In comparison with free-living fungi, A. areolatum needs only to adapt to a relatively simple living environment; it grows inside the xylem of weaker pine stems and is transferred by its symbiotic insect hosts. In our results, the symbiotic fungus had a large number of carbohydrate enzyme genes but few virulence and transporter genes. This was consistent with their symbiotic relationship; A. areolatum provides nutrients for the growth of S. noctilio larvae by secreting extracellular enzymes, and S. noctilio protects and transfers the fungus before introducing into a new suitable host substrate by carrying the fungus inside a specialized organ, the mycangium.

### Sesquiterpene synthase clusters.

Female woodwasps are more likely to be attracted by trees that are weakened or that had been attacked previously (21). Volatile components are critical factors that are necessary for S. noctilio to locate suitable hosts and oviposition spots in different ranges (22). Studies have shown that mated female woodwasps were more attractive to the volatile components of A. areolatum than to those of the plant hosts; sesquiterpene compounds may play a role in this process (24). An analysis of sequence data from Russulales showed that sesquiterpene synthase homologs were widespread among these fungi. However, despite the preponderance of sesquiterpene synthase homologs in fungi, relatively little is known about their activities and biological functions. Cloning and characterization of sesquiterpene synthases of C. cinereus to the Escherichia coli expression vector revealed that α-muurolene, β-elemene, γ-muurolene, germacrene D, and δ-cadinene were produced by Cop3 cultures. δ-Cadinene, β-cubebene, sativene, β-copaene, and cubebol were detected in the headspace of Cop4 cultures (24). We speculated that the same compounds would appear in the volatile profiles of A. areolatum. What sesquiterpene components are main factors that enable S. noctilio to locate the hosts have been attacked previously needs further study, and the results would be important in developing effective lures for this pest.

The A. areolatum genome is the first reported genome sequence of a woodwasp symbiotic fungus. The genomic resources presented here, including the genome sequences and annotations, as well as the detailed lists of cell wall-degrading enzymes, virulence-associated genes, and sesquiterpene synthase clusters show that the symbiotic fungus can release chemicals, attracting more female woodwasps to oviposit, and can degrade plant cell wall components by secreting cellulase, hemicellulose, and ligninase, which provide S. noctilio with a suitable environment and nutrients for the larval growth. These results lay a foundation for our understanding of the mechanism of this combined fungus-insect damaging system.

## MATERIALS AND METHODS

### Strains and culture conditions.

In 2017, A. areolatum was isolated from S. noctilio females collected from Mongolian pines (P. sylvestris var. mongolica) in Jun De Forest Farm (130°17′47″E, 47°12′11″N), Hei Longjiang Province, China. The fungus strain was confirmed as A. areolatum by morphological characteristics and molecular analysis in the internal transcribed spacer (ITS) and large subunit (LSU) regions as described previously by Wang et al. (15). The fungi were cultured on potato dextrose agar (PDA) and preserved at the Beijing Key Laboratory for the Control of Forest Pest, Beijing Forestry University, Beijing, China. For genomic DNA extraction, the fungi were cultured on PDA plates for 2 weeks at 25°C. Mycelia were collected in sterile tubes, washed with sterile water, immediately frozen with liquid nitrogen, and stored at −80°C until used.

### Genomic DNA extraction and sequencing.

The total genomic DNA of A. areolatum was extracted based on an improved cetyl trimethyl ammonium bromide (CTAB) procedure (51) and was sequenced using the Illumina HiSeq 4000 and PacBio Sequel platforms. For second-generation sequencing on the HiSeq 4000 platform, a large fragment library was prepared using a TruSeq DNA PCR-Free library prep kit (catalog no. FC-121-3001; Illumina, San Diego, CA, USA) with an average insertion size of 270 bp. The library was quantified using an Agilent 2100 bioanalyzer instrument (Agilent DNA 1000 reagents; Agilent, Santa Clara, CA, USA) and real-time quantitative PCR (RT-qPCR). The qualified libraries were amplified within the flow cell on the cBot instrument for cluster generation (HiSeq 4000 PE cluster kit; Illumina). The clustered flow cell was loaded onto a HiSeq 4000 sequencer for paired-end sequencing (HiSeq 4000 SBS kit; Illumina) with recommended read lengths of 150 bp. For third-generation sequencing on a PacBio Sequel platform, genomic DNA samples were sheared to >10 kb using g-Tube (Covaris, Woburn, MA, USA). Libraries were prepared using SMRTbell template prep kit 1.0 (code 100-259-100; Pacific Biosciences, Menlo Park, CA, USA) with BluePippin size selection for fragments of >7 kb. The resulting libraries were sequenced on the PacBio Sequel platform at the Beijing Genomics Institute (Shenzhen, Guangdong, China).

### Genome assembly.

The Illumina sequencing produced 6,839 Mb of clean data from 7,784 Mb of raw data using SOAP *de novo* technology. The low-quality reads obtained by PacBio sequencing were filtered using SMRT Analysis v. 2.3.0 (52), and the subreads were subjected to two rounds of error correction using Falcon v. 0.3.0 and Proovread 2.12. The corrected PacBio reads were assembled with Celera Assembler 8.3 and Falcon v. 0.3.0. The Illumina sequences were used for k-mer analysis (Fig. S1) and correcting the PacBio assembly. To obtain a high-quality assembly, the sequence errors were corrected by GATK v1.6-13. Then, the contigs were combined into scaffolds using the SSPACE_Basic v. 2.0 tool (53) and the gaps were closed using PBJELLY 15.8.24. Finally, we applied a redundant pipeline to identify and remove the heterozygous contigs and scaffolds, based on pairwise sequence similarity searches (54). The assembly candidate was evaluated by the use of BUSCO v 3.0.2 (55) with the data set from the fungus lineage.

10.1128/mSphere.00301-20.1 FIG S1 Frequency distribution of 15-mers in the genome sequencing reads. Occurrences of 15-mer DNA sequences were counted in a total of 7,784 Mb of HiSeq 4000 reads. Inflated k-mers in low-frequency ranges originated from sequence errors. The main peak was found at a frequency of 30 counts. This might come from homozygous segments of the genome. A faint peak located at around a frequency of 15 counts may represent the heterozygosity sequence. Genome size was estimated by dividing the total k-mer count (1,299.51 M) by the main peak depth (30.02 counts), giving a size for *A. areolatum* genome of 43.28 M. Download FIG S1, TIF file, 0.1 MB. Copyright © 2020 Fu et al. 2020 Fu et al. This content is distributed under the terms of the Creative Commons Attribution 4.0 International license.

10.1128/mSphere.00301-20.2 FIG S2 Phylogenetic analysis of putative ABC transporters of A. areolatum and other fungi of Agaricomycotina. Coprinopsis cinerea ABC transporter designations (GenBank accession numbers) are as follows: CcABCA1 (EAU92463); CcABCB1 (EAU89622); CcABCB2 (EAU86002); CcABCB3 (EAU80745); CcABCB4-1 (EAU81212); CcABCB4-2 (EAU81213); CcABCB5 (EAU81372); CcABCB6 (EAU80415); CcABCB7-1, CcABCB7-2, and CcABCB8 (EAU91240); CcABCB9 (EAU83155); CcABCB10 (EAU91355); CcABCB11 (EAU92413); CcABCB12 (EAU84325); CcABCB13 (EAU80676); CcABCB14 (EAU86903); CcABCB15 (EAU86911); CcABCB16 (EAU86922); CcABCC1 (EAU92643); CcABCC2 (EAU87480); CcABCC3 (EAU85519); CcABCC4 (EAU86771); CcABCC5 (EAU86674); CcABCC6 (EAU89464); CcABCC7 (EAU89473); CcABCC8 (EAU89511); see_CcABCC12 (EAU86556); CcABCC9 (EAU90288); CcABCC10 (EAU92544 plus EAU92545); CcABCC11 (EAU85371 plus EAU85372); CcABCC12-1, CcABCC12-2, and CcABCD1 (EAU92657); CcABCD2 (EAU82829); CcABCE1 (EAU89439); CcABCF1 (EAU91504); CcABCF2 (EAU92935); CcABCF3 (EAU81879); CcABCF4 (EAU82547); CcABCF5 (EAU82216); CcABCG1 (EAU92343); CcABCG2 (EAU85334); CcABCG3 (EAU81966); CcABCG4 (EAU8722); CcABCG5 (EAU89680); CcABCG6 (EAU83316); CcABCG7 (EAU81185 plus EAU81186); CcABCI1 (EAU84765); CcABCI2 (EAU91779); and CcABCI3 (EAU88531). Cryptococcus neoformans GenBank accession numbers (ABC transporter designations) are as follows: AAW41302 (CnABCB1), AAW41802 (CnABCB2), AAW41541 (CnABCB3), AAW45126 (CnABCB4), AAW45354 (CnABCB5), AAW46499 (CnABCB6), AAW41299 (CnABCC1), AAW41361 (CnABCC2), AAW42094 (CnABCC3), AAW42503 (CnABCC4), AAW43405 (CnABCC5), AAW43689 (CnABCC6), AAW44522 (CnABCC7), AAW44521 (CnABCC8), AAW45454 (CnABCC9), AAW41159 (CnABCD1), AAW42317 (CnABCD2), AAW41230 (CnABCE1), AAW40703 (CnABCF1), AAW43068 (CnABCF2), AAW42954 (CnABCF3), AAW45788 (CnABCF4), AAW41207 (CnABCG1), AAW41688 (CnABCG2), AAW43038 (CnABCG3), AAW44322 (CnABCG4), AAW45244 (CnABCG5), AAW46652 (CnABCG6), AAW47087 (CnABCG7), AAW47099 (CnABCG8), AAW43875 (CnABCI1), AAW45396 (CnABCI2), and AAW46280 (CnABCI3). Download FIG S2, TIF file, 1.5 MB. Copyright © 2020 Fu et al. 2020 Fu et al. This content is distributed under the terms of the Creative Commons Attribution 4.0 International license.

10.1128/mSphere.00301-20.3 FIG S3 The terpenoid gene clusters of A. areolatum. The figure displays 20 neighboring genes surrounding terpene synthase genes. Each gene is labeled with a pfam annotation when available. Identical genes are marked with the same color. Download FIG S3, TIF file, 0.1 MB. Copyright © 2020 Fu et al. 2020 Fu et al. This content is distributed under the terms of the Creative Commons Attribution 4.0 International license.

### Gene prediction and annotation.

Protein-coding genes were predicted through a combination of *de novo* prediction and transcriptome-based prediction methods. For *ab initio* predictions, SNAP v. 2010-07-28 (56), Augustus v. 3.2.1 (57), and GeneMark ES v. 4.21 (58) were used to predict coding genes. Then, transcriptome sequencing (RNA-Seq) data (unpublished) were mapped to the assembly using Tophat v2.0.8 and the transcripts were assembled to gene models by the use of Cufflinks v2.1.1 (59). Finally, all gene models predicted by the methods named above were combined into a nonredundant set of gene structures by the use of EVidenceModeler (EVM) (60). The tRNA regions and secondary structures were detected using tRNAscan-SE v. 1.3.1 (61). The rRNAs were analyzed using RNAmmer software, and the small RNAs (sRNAs) were predicted using Infernal to search against the Rfam (v. 9.1) database (62, 63). The predicted gene models were functionally annotated by the use of BLASTp searches against the National Center for Biotechnology Information nonredundant database, Swiss-Prot (64), TrEMBL, Cluster of Orthologous Groups (COG) (65), Gene Ontology (GO) (66), and Kyoto Encyclopedia of Genes and Genomes (KEGG) (67, 68).

### Transposable elements and tandem repeat identification.

To evaluate the TEs within the A. areolatum genome, a sequence alignment prediction method and a *de novo* prediction method were used. For the sequence alignment prediction method, the TEs were searched with the supporting database Repbase using RepeatMasker v. 4-0-6 (http://www.repeatmasker.org/) (69). The optimized default parameters were utilized with the “-lib” option to find repeats, and the “-species Fungi” option was applied in a separate analysis to find fungal repeats. Repeat Protein Masker and the transposon protein library associated with RepeatMasker were also used to identify TEs. The *de novo* prediction method was used firstly to produce an eXtended Database Format (XDF) using buildXDFDatabase. Then, TE models were established using Repeat Modeler with the XDF database and these models were then used to predict TEs using Repeat Masker. Tandem repeats were evaluated using Tandem Repeat Finder v. 4.04 (http://tandem.bu.edu/trf/trf.html) (70).

### Phylogenetic analysis.

The genome sequences of 79 Agaricomycetes species (see Table S4 in the supplemental material) were downloaded from the NCBI database in FASTA format. A group of orthologous genes (phylogenetically conserved) in the fungal genome were obtained by the use of OrthoFinder 2.2.7 (71). We selected 41 single-copy orthologs to build the phylogenetic tree. Sequence alignment was done using MAFFT 7.409, and the conserved sites were extracted and concatenated by the use of Gblocks 0.91b. We ran ProtTest v2.0 to select the most appropriate model and used RAxML 8.2.12 (72) to build the ML gene tree with the “-f a -x 12345 -p 12345 -m PROTGAMMAILGF -N 1000” options (73). Wallemia sebi (GenBank accession number GCF_000263375.1) protein sequences were used as an outgroup (74, 75).

### CAZyme analysis.

CAZymes in A. areolatum and 10 other fungi were identified and annotated using BLAST (76), and the dbCAN annotation program HMMER 3 (http://bcb.unl.edu/dbCAN2/index.php) was used to search against the CAZy (carbohydrate-active enzyme) database (v. 2017-09; http://www.cazy.org/) (77, 78). The results were combined when the E value was less than 1E−05. The class II peroxidases and DyPs (dye-decolorizing peroxidases) were further confirmed by BLAST searches against the PeroxiBase database (http://peroxibase.toulouse.inra.fr/) (79).

### Prediction of virulence-related factors.

Candidate virulence-associated genes were identified within the A. areolatum genome using BLASTp to search against PHI-base v. 4.3 (http://www.phi-base.org/) (20). Protein alignments were performed to identify putative virulence-associated genes in A. areolatum with more than 40% identity and 70% query coverage (80). The virulence factors (VFs) were searched using BLAST against the Virulence Factor Database (VFDB; http://www.mgc.ac.cn/VFs/) (81). The Transporter Classification Database (TCDB) contains sequences, classifications, and structural, functional, and evolutionary information about transport systems from a variety of taxa (82). Candidate transporters in A. areolatum were identified based on searches of the Transporter Classification Database (TCDB; http://www.tcdb.org/) with an E value threshold of 1E−05 and identity values of >40% (83). The sequence alignment of ABC transporters of fungi of Agaricomycotina was performed in MAFFT 7.409, and the phylogenetic tree was built using FastTree 2.1.7.

### Secondary metabolism gene prediction.

The secondary metabolism biosynthesis genes and gene clusters were predicted with AntiSMASH 5.0.0 (84) in the genomes of A. areolatum, S. hirsutum, *Peniophora* sp. strain CONT, and H. irregulare. Putative fungal terpene synthase sequences of 83 Agaricomycetes genomes were obtained in two ortholog groups. Five sesquiterpene synthase homologues (named Cop1 to Cop5) identified in C. cinereus were also included. For phylogenetic tree construction, alignments were manually inspected to exclude sequences that seemed to be incorrectly annotated (e.g., sequences that appeared to be too short or too long). The sequence alignment of 902 terpene synthase genes was performed in MAFFT 7.409, and the phylogenetic tree was built using FastTree 2.1.7.

### Data availability.

The genome assembly has been deposited in the NCBI/DDBJ/GenBank genome database under accession number SAXG00000000. The A. areolatum raw sequence data have been deposited in the NCBI database under BioProject accession number PRJNA513942 and BioSample accession number SAMN10716400.
