# Supplementary figures and images for: Genome Sequencing and Analysis of the Fungal Symbiont of Sirex noctilio, Amylostereum areolatum: Revealing the Biology of Fungus-Insect Mutualism
Source: mSphere. 2020 May 13;5(3):e00301-20. doi: 10.1128/mSphere.00301-20 (PMC7227769; doi:10.1128/mSphere.00301-20)

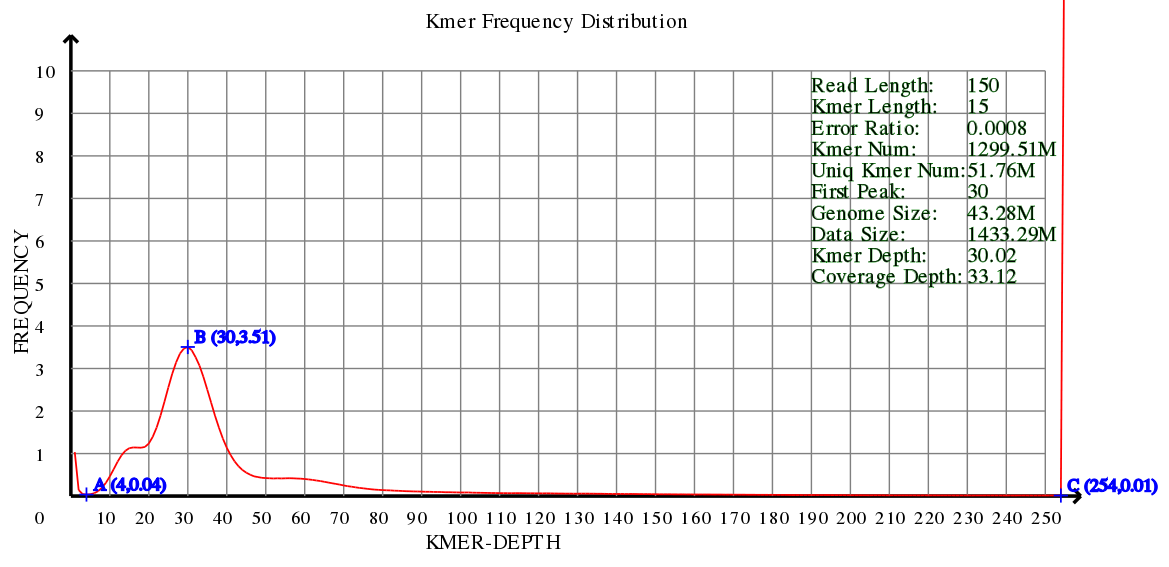

Supplement: FIG S1 [file mSphere.00301-20-sf001.tif]

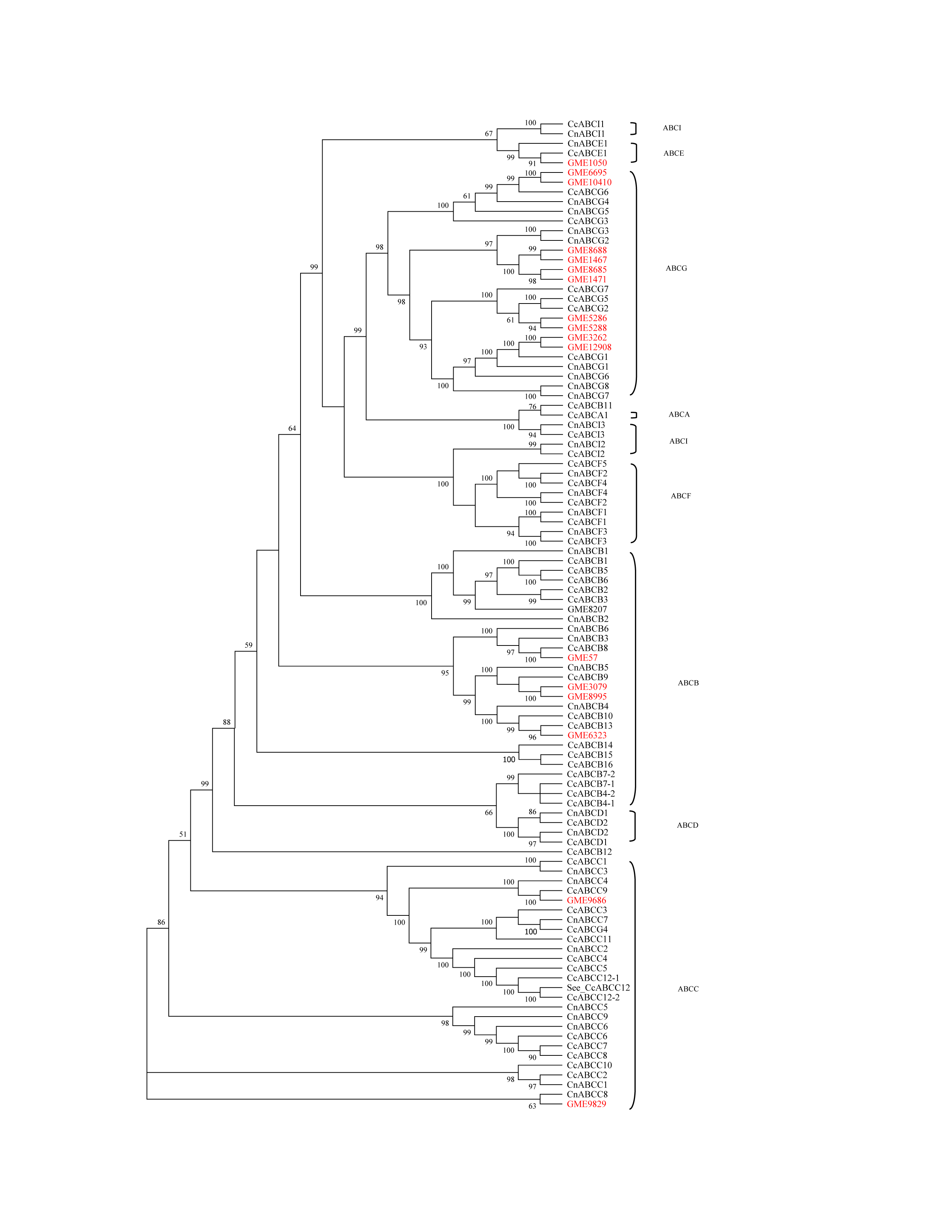

Supplement: FIG S2 [file mSphere.00301-20-sf002.tif]

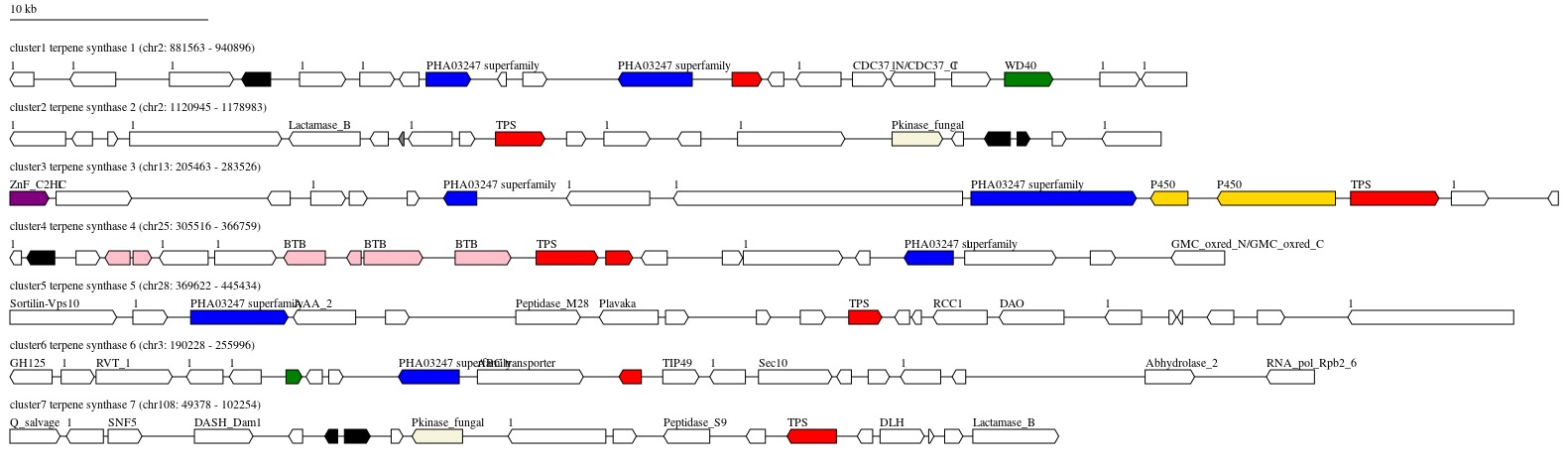

Supplement: FIG S3 [file mSphere.00301-20-sf003.tif]
